# Expectant management of traumatic intussusception

**DOI:** 10.1093/jscr/rjaa594

**Published:** 2021-01-31

**Authors:** Kate Swift, Krish Kulendran

**Affiliations:** Department of Surgery, Cairns Base Hospital, Cairns City, Queensland, Australia; Department of Surgery, Cairns Base Hospital, Cairns City, Queensland, Australia

## Abstract

Mesenteric injuries and traumatic intussusception are rare surgical presentations following blunt trauma, with potentially life-threatening complications. Diagnosis relies on high clinical suspicion and judicious use of imaging in trauma. Literature suggests that these presentations should always be managed operatively for diagnostic clarity, manual reduction of intussusception and, if indicated, resection of involvement segment. However, in the setting of a stable patient with a reassuring examination, this may not be necessary. This case presents the successful expectant management of a traumatic mesenteric haematoma acting as a pathologic lead point for small bowel intussusception.

## INTRODUCTION

Traumatic intussusception is an exceedingly rare presentation, either in isolation or in association with mesenteric injury. In the existing literature, this is managed operatively with manual reduction or resection of the involved bowel segment. The authors present a case of blunt abdominal trauma in which mesenteric haematoma acted as a pathological lead point for entero-enteric intussusception. The patient was successfully managed conservatively on the basis of their clinical features, with an excellent outcome.

## CASE REPORT

**Figure 1:**
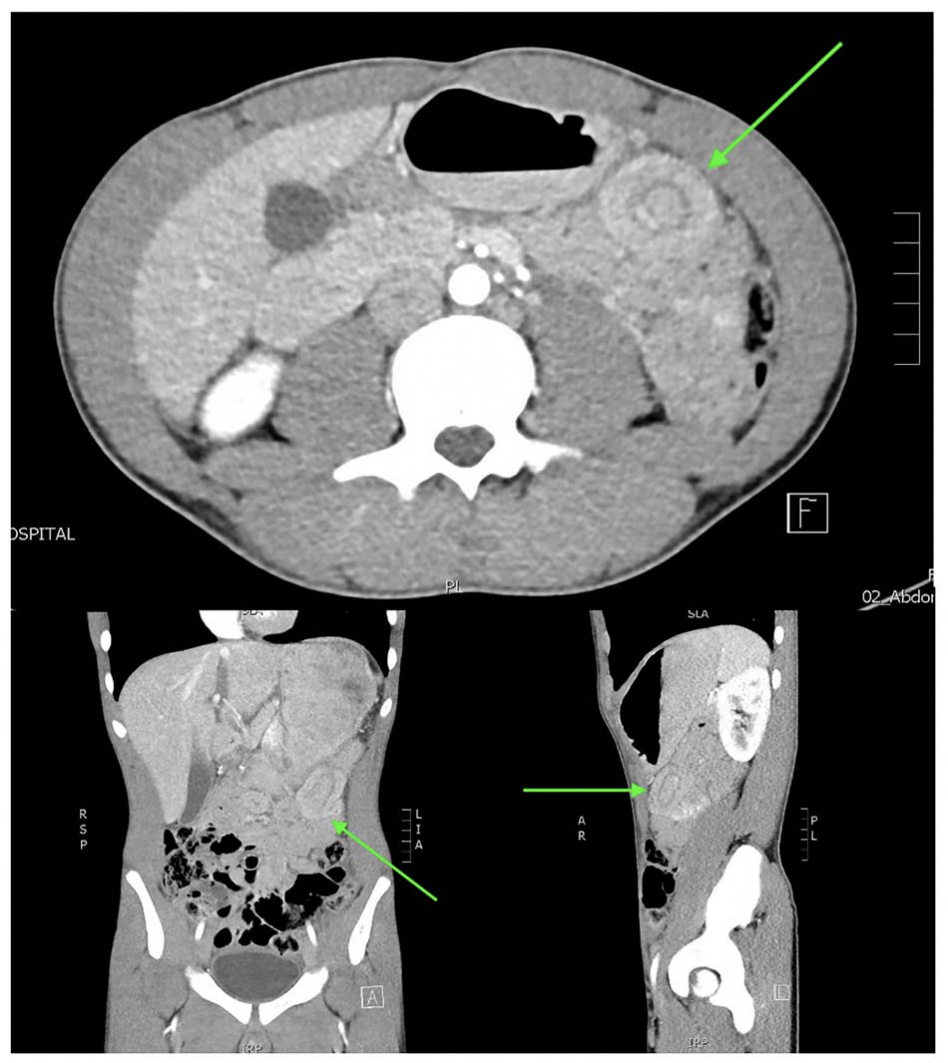
Abdominal CT in axial, coronal and sagittal planes demonstrating mesenteric haematoma and jejuno-jejunal intussusception (‘bowel-within-bowel’ sign indicated by green arrow).

A normally fit and well 15-year-old male presents after being pinned between a tree and a car at low speed. He has no loss of consciousness and self-extricates on scene. On arrival, he is alert and haemodynamically stable. He has minor bruising to his upper abdomen and no other external injuries. His abdominal examination is reassuring with no peritonism. His biochemistry is normal with a haemoglobin of 143 g/L and a lactate of 1.2 mmol/L. An abdominal focussed assessment with sonography in trauma (FAST) scan is positive for free fluid. The patient remains stable and proceeds to contrast-enhanced abdominal computed tomography (CT), which reveals small volume haemoperitoneum and a hyperdense focus 45 × 31 × 30 mm between bowel loops left mid-abdomen in keeping with interloop mesenteric haematoma, abutting the base of a 5 cm length of entero-enteric intussusception (Fig. 1). Adjacent small bowel loops are thick walled, with no evidence of obstruction or perforation.

Given his reassuring biochemistry and clinical examination, and with a known benign lead point, the patient is managed conservatively with bowel rest and serial abdominal examinations. He remains pain free for the duration of his hospital stay, and his follow-up haemoglobin and venous gas are unchanged. Tertiary survey reveals no new injuries. He is subsequently discharged from hospital after 48 h of observation, having tolerated a slow diet upgrade and passed a normal bowel motion. He has no abdominal complaints at follow-up 5 months post-injury.

## DISCUSSION

Traumatic mesenteric haematoma is a rare sequelae of blunt abdominal trauma, representing <1% of all injuries [1]. A review of gastrointestinal tract injuries in the Westmead trauma database over a 12-year period identified only three cases of mesenteric haematoma, from a blunt trauma cohort of 22 870 patients [2]. Haematoma develops due to direct crush injury, with bowel being compressed against the bony vertebrae causing mural or mesenteric injury, or due to shearing forces during rapid deceleration [2]. Mesenteric injuries are notoriously difficult to identify clinically and can lead to morbid complications, including devitalized bowel, delayed perforation, obstruction and post-traumatic stenosis/stricture.

Traumatic intussusception is also exceedingly rare. Intussusception is the ‘telescoping’ of bowel along with its mesentery into the distal lumen, propagated by peristalsis. If left untreated, this segment of bowel can obstruct its vascular supply and become necrotic, resulting in peritonitis. The aetiology of intussusception differs in the paediatric and adult population. Children present relatively commonly, with 90% of cases occurring earlier than 2 years of age; these are usually idiopathic or post-viral, without underlying sinister pathology [3]. Adults present rarely and less classically, either with symptoms of obstruction or intermittent abdominal pain. They have an underlying pathological cause in up to 90% of cases (e.g. malignancy or tumour, inflammation, Meckel’s diverticulum) and routinely require surgical resection for diagnosis and management [4]. CT is described as the imaging modality of choice, with a classical appearance of concentric rings of ‘bowel-within-bowel’ (equivalent to the ‘target sign’ on ultrasound). However, the majority of cases are diagnosed intraoperatively on direct visualization.

There is a paucity of literature describing traumatic intussusception in adults; these most commonly had a delayed presentation with predominance of obstructive symptoms [5], and all were managed operatively. A broad literature review in *The Journal of Trauma* in 2005 identified only 17 published cases of intussusception following blunt trauma, two of which were associated with haematomas [6]. In the absence of mesenteric haematoma as a lead point, traumatic intussusception has been attributed to local smooth muscle spasm, impaired peristalsis and bowel wall oedema [5]. Manual reduction without resection is cited as the preferred management of traumatic intussusception in both adults and children [6]. Inappropriate delay in diagnosis and management places a patient at risk of bowel ischaemia and perforation or ongoing or delayed haemorrhage (particularly, as vasospasm relaxes) [7].

**Figure 2:**
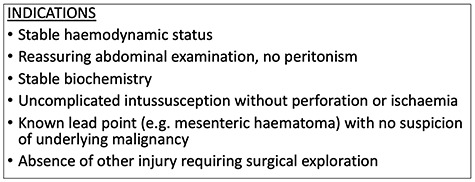
Proposed indications for conservative management of traumatic intussusception.

This patient was managed expectantly without need for surgical exploration and did not suffer any immediate or delayed complications. In an otherwise healthy adolescent without suspicion of an intraluminal pathological lead point, this represents the best possible outcome. Whilst the body of literature suggests that traumatic mesenteric haematoma and associated intussusception should undergo operative reduction, an argument can be made for conservative management with vigilant monitoring, with hope of spontaneous resolution. Proposed indications for conservative management are outlined in Fig. 2. Deterioration or failure to improve should prompt urgent surgical exploration. The take-home message for clinicians is that patients can potentially be safely managed conservatively on the merit of their clinical signs and symptoms, rather than their imaging findings.

## CONFLICT OF INTEREST STATEMENT

None declared.

## FUNDING

None.

## CONSENT

Informed consent was obtained from the patient and their legal guardian for publication and use of accompanying images. Case details have been de-identified to protect patient anonymity.
